# Post-discharge healthcare utilization and costs in musculoskeletal surgery patients: A cohort study in Korea

**DOI:** 10.1371/journal.pone.0342252

**Published:** 2026-02-06

**Authors:** Boyoung Jeon, Boyoung Jung, Yun-Kyung Song

**Affiliations:** 1 Department of Health and Medical Information, Myongji College, Seodaemun-gu, Seoul, Republic of Korea; 2 Department of Health Administration, Hanyang Women’s University, Seoul, Republic of Korea; 3 Department of Korean Rehabilitation Medicine, College of Korean Medicine, Gachon University, Seongnam-si, Republic of Korea; Yonsei University Medical Center: Yonsei University Health System, KOREA, REPUBLIC OF

## Abstract

**Background:**

Musculoskeletal surgery imposes extended recovery periods and significant financial burdens that can undermine individual and system-level health security. Patients undergoing musculoskeletal surgery often face prolonged recovery and substantial post-discharge costs, yet longitudinal evidence on their healthcare use remains limited.

**Methods:**

This study quantified two-year post-discharge utilization and identified predictors of high expenditure among Korean musculoskeletal surgery patients. A retrospective cohort was constructed from the 2019–2021 Korea Health Panel. Adults (n = 182) hospitalized for spinal, knee, shoulder or other musculoskeletal disorders between July 2019 and June 2020 were followed for 24 months. Outcomes were total healthcare expenditure (log-transformed) and in the top 25% cost group (“high-expenditure”) in the second post-discharge year.

**Results:**

Among 182 adults hospitalized for musculoskeletal surgery, first-year post-discharge spending averaged US $848 but fell to US $487 in the second year. Readmission fell from 19.2% to 7.1%, and Western-medicine outpatient visits declined from 18.3 ± 25.9 to 13.6 ± 22.9 per person. By contrast, Traditional Korean Medicine (TKM) visits rose from 2.3 ± 6.5 to 3.3 ± 10.0. In multivariable models, metropolitan residence, obesity, additional chronic conditions, and heavier first-year inpatient and outpatient use independently predicted higher second-year costs. Lower household income was associated with lower spending. Index diagnoses were pivotal: spinal disorders and shoulder disorders markedly increased the odds of falling into the top-cost quartile. Among the first-year TKM, frequent chuna/manual therapy sessions were marginally associated with higher costs, suggesting these rehabilitative modalities may serve as proxies for underlying health complexity during the stabilization phase.

**Conclusions:**

Spinal and shoulder disorders, metropolitan residence, obesity, multimorbidity, heavy inpatient and outpatient use during the first post-discharge year, and frequent TKM sessions, albeit marginally, jointly predicted the highest second-year expenditures. These findings highlight the value of early risk stratification and tightly coordinated Western-and-traditional care pathways that facilitate the shift from structural repair to functional restoration. From a policy perspective, these results suggest the need for integrated post-discharge care models and targeted financial support strategies to reduce avoidable costs and enhance equity in musculoskeletal rehabilitation.

## 1. Introduction

Musculoskeletal impairments refer to health conditions characterized by temporary or permanent limitations in physical function and participation in daily activities due to damage in the muscles, bones, joints, and adjacent connective tissues [1], affecting all age groups with spinal/joint disease diagnosis age decreasing amid rising life expectancy (global: 73.4 years; South Korea: 84.4 years) and lifestyle changes [2,3]. These conditions, affecting all age groups with decreasing diagnosis age for spinal/joint diseases amid aging and lifestyle changes, commonly arise from occupational risk factors and can progress to chronic disability without proper management [2,4,5].

Musculoskeletal disorders are among the most frequently treated surgical conditions in South Korea, ranking highly in both surgical volume and healthcare expenditures [6,7]. For example, in 2022, knee osteoarthritis surgeries (M17, according to the Korean Classification of Diseases [KCD], which is adapted from the International Classification of Diseases [ICD]) alone cost 676.3 billion KRW, with spinal and pelvic procedures also ranking high in volume and expenditure [8]. It imposes substantial economic burden, estimated at 0.7% of GDP including direct costs and productivity losses [6]. Post-discharge care after musculoskeletal disorders surgery entails substantial healthcare resource use and financial burden, including rehabilitation, pain management, and readmission risks. High-cost users (top 5–10%) account for over 50% of expenditures; early prediction enables targeted case management, reducing readmissions and enhancing National Health Insurance sustainability [9].

In South Korea, policymakers now regard seamless post-discharge continuity of care as a cornerstone of national health security [9]. An evaluation of the 2019–2022 Integrated Medical–Social Care pilot found that nearly one in five hospitalizations after musculoskeletal surgery could have been avoided. The Integrated Community Care Assistance Act, enacted in 2024, mandates home-based rehabilitation, multidisciplinary outreach, and financial protection for high-risk surgical populations, signaling a systemic shift from institution-centered to community-centered recovery pathways [10]. However, empirical evidence remains scarce on longitudinal post-discharge healthcare cost trajectories after musculoskeletal surgery in Korea, and little is known about which patient and service factors drive high post-surgical expenditure [11].

Therefore, this study aims to characterize longitudinal post-discharge healthcare cost trajectories after musculoskeletal surgery in Korea and to identify key factors associated with high healthcare expenditure. Specifically, it investigates the changes in healthcare service usage during the 6, 12, 18, and 24 months following discharge and classifies patients into distinct cost trajectory groups. Additionally, the study examines how healthcare utilization in the first post-discharge year is associated with high-cost trajectories and expenditure levels in the second year.

## 2. Materials and methods

### 2.1. Data source and study population

This study utilized data from the second cohort (2019–2021) of the Korea Health Panel (KHP), which was jointly provided by the Korea Institute for Health and Social Affairs (KIHASA) and the National Health Insurance Service (NHIS). To ensure a consistent 24-month follow-up period for all participants within the available dataset, a retrospective cohort was constructed by identifying patients hospitalized for musculoskeletal disorders between July 2019 and June 2020 as the index admission period. This allowed us to track each patient’s healthcare utilization for exactly two years following their discharge date.

This retrospective cohort study analyzed patients admitted for medical treatment involving surgical procedures. To define musculoskeletal disorders, we utilized three primary diagnosis codes from both Western Medicine (WM) and Traditional Korean Medicine (TKM) and reclassified these into four groups based on diagnosis names [12]. These specific conditions—including spinal, knee, and shoulder disorders—were selected because they represent the highest surgical volume and healthcare expenditures within the Korean National Health Insurance system [6-8]. To ensure clinical relevance and maintain statistical power, musculoskeletal disorders were reclassified into four distinct groups: 1) spinal disorders, 2) knee osteoarthritis, 3) shoulder joint disorders, and 4) other joint/muscle pain, fractures, and gout. The fourth group, while heterogeneous, was combined due to sample size limitations to ensure the analysis focused on high-volume diagnostic categories consistently identified in prior economic burden studies [6-8].

In cases where the codes varied by year, the diseases were reclassified based on their names into four groups: spinal disorders, knee osteoarthritis, shoulder joint disorders, and other joint pain, muscle pain, fractures, and gout. “Spinal disorders” included intervertebral disc diseases and other spinal diseases (such as spinal stenosis, vertebral compression fractures, scoliosis, and spondylitis). “Knee osteoarthritis” included knee degenerative arthritis and knee joint replacement surgeries. “Shoulder joint disorders” included frozen shoulder, rotator cuff disorders, and calcific tendinitis. “Other fractures, knee-related osteoarthritis (degenerative arthritis), other joint pain and muscle pain (sprains, contusions, etc.), and gout” were defined as one group due to sample size limitations.

An “inpatient episode” was defined as a continuous hospitalization at the same healthcare institution where the interval between claims did not exceed one day. If the interval between claims exceeded 14 days, even at the same institution, it was considered a new episode. In cases where a patient was readmitted within 14 days after surgery, the last admission was considered as the index admission for follow-up [12,13]. This 14-day readmission rule was utilized to ensure the index admission captured the complete surgical episode and is aligned with HIRA guidelines for episode grouping [13].

Based on these criteria, we initially identified 521 inpatient cases of musculoskeletal disorders between 2019 and 2020. We excluded 2 duplicate claims for the same episode and 17 cases of readmission within 14 days after surgery. In addition, 269 cases were excluded due to admission dates falling outside the defined observation window (i.e., before July 2019 or after June 2020), which were not eligible for full follow-up.

**Fig 1:**
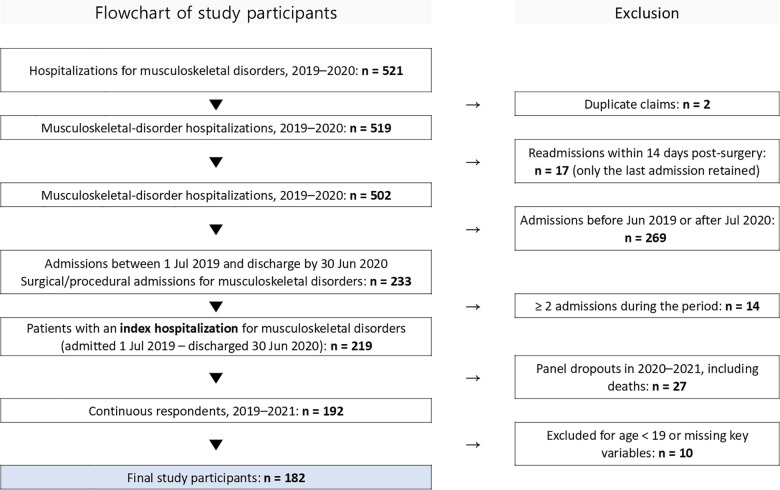
Flow diagram of participant selection.

To construct a person-level dataset, 14 patients with multiple admissions during the target period were excluded. As a result, 219 patients who were admitted between July 1, 2019, and June 30, 2020, were included. From this group, 27 individuals were excluded due to death or panel dropout during the follow-up period (2020–2021), and 10 individuals were excluded for being under 19 years old or having missing values in key variables. The final analytic sample comprised 182 patients. No imputation methods were applied to handle missing data, instead, this study employed a complete-case analysis approach, whereby only participants with full data for all covariates and outcome variables were included in the final analytic sample to maintain the consistency of the multivariable estimations [14] (Fig 1).

### 2.2. Definition of variables

#### 2.2.1. Outcome variables.

The primary outcome variables in this study are “log(total healthcare expenditures)” at the second year after discharge (366–730 days from discharge), and the “high-expenditure” at the second year. Total healthcare expenditures are the sum of the amounts paid by the patient to healthcare institutions, including payments for prescriptions, and are recorded as 0 when no expenditure occurred. This amount includes both covered and non-covered expenses under health insurance, representing the amount the patient directly pays to the healthcare institution. The total healthcare expenditure was log-transformed to normalize the typically right-skewed distribution of medical cost data and to satisfy the assumptions of the linear regression model by minimizing the influence of extreme outliers [15].

High-expenditure is defined as being in the top 25% of total healthcare expenditures for each year [15]. Based on the threshold for the top 25% of total healthcare expenditures, high-expenditure in the first year after discharge was defined as healthcare expenditures of 1,017,360 KRW (≈ US $859.6, 2020 dollars) or more, and high-expenditure in the second year after discharge was defined as 551,700 KRW (≈ US $466.1, 2020 dollars) or more (1 USD = 1 184.1 KRW). To analyze the binary high-expenditure outcome, we used a multivariable logistic regression model formally defined as logit(P) = ln(P/(1-P)) = β0 + ∑βiXi, where P is the probability of a patient falling into the top 25% expenditure group and Xi represents the set of patient characteristics and healthcare utilization factors [16].

#### 2.2.2. Explanatory variables.

The main explanatory variables are the characteristics of the index hospitalization and the characteristics of healthcare utilization within one year after discharge. The characteristics of the index hospitalization include the primary diagnosis, the location of the medical institution, the type of medical institution, and the type of medical institution the patient first visited after discharge. The primary diagnosis refers to the condition diagnosed during the index hospitalization, and it falls into one of the four categories previously described (spine-related disorders, knee osteoarthritis, shoulder joint disorders, and other joint pain, muscle pain, fractures, and gout). The location of the medical institution is categorized into the metropolitan area (Seoul, Gyeonggi, Incheon) and non-metropolitan areas. The type of medical institution is classified into clinics, hospitals, general hospitals, and tertiary general hospitals. Additionally, the first healthcare facility the patient visited after discharge is categorized into no healthcare utilization, clinics, hospitals, general hospitals, and tertiary general hospitals. In regression analysis, “no healthcare utilization” and “clinic-level” were combined into one category.

The variables of healthcare utilization within one year after discharge were included as explanatory variables for healthcare expenditure in the second year post-discharge. Healthcare utilization variables from the first year post-discharge—specifically the frequency of inpatient admissions and outpatient visits to both medical and traditional medicine clinics—were included as explanatory variables to predict expenditures in the second year [17]. TKM modalities were disaggregated into three categories based on distinct treatment goals for musculoskeletal recovery: 1) acupuncture-related services (moxibustion, cupping, and herbal acupuncture) primarily for pain relief; 2) chuna, manual, and physical therapy for functional rehabilitation and musculoskeletal correction; and 3) herbal medicine (decoctions and expensive formulas) for constitutional recovery [12].

These variables included the number of inpatient admissions, outpatient visits to medical institutions, and outpatient visits to traditional medicine clinics. During the follow-up period, there were no cases of inpatient admissions in TKM, so only medical institution admissions were included. For TKM, Model 1 used the number of outpatient visits to traditional medicine clinics, and Model 2 utilized the frequency of each of the three types of TKM (’acupuncture, moxibustion, cupping, herbal acupuncture,’ ‘decoctions, expensive medicines, general herbal medicine,’ and ‘chuna therapy, manual therapy, physical therapy’). Acupuncture, moxibustion, cupping, and herbal acupuncture are TKM primarily aimed at pain relief. Chuna therapy, manual therapy, and physical therapy are rehabilitative treatments primarily for musculoskeletal correction and recovery. Decoctions, expensive medicines, and general herbal medicine are pharmacological treatments aimed at strengthening the body constitution. The classification of TKM was grouped according to the treatment goals for musculoskeletal disorders.

#### 2.2.3. Covariates.

The other personal characteristics variables were based on data from the same year as the index hospitalization. The demographic characteristics included gender (male, female), age (continuous variable), marital status (married, unmarried), and residential area (metropolitan area, non-metropolitan area). Socioeconomic characteristics included income level (annual income quartiles), type of health insurance (National Health Insurance, Medical aid), and job status (economically inactive, wage workers, employers and self-employed). These socioeconomic covariates were included because they are critical determinants of healthcare seeking behavior and the ability to afford non-covered medical expenses in the Korean healthcare system, which often interact with clinical factors to shape the overall financial burden [18-20].

Additionally, variables representing individual health behaviors and health status included regular exercise (exercise, no exercise), smoking (current smoker, non-smoker), binge drinking (binge drinking in the past year, none), obesity status (BMI ≥ 25 kg m², BMI < 25 kg m²), health conditions (having health issues in the past year, none), number of chronic conditions, health-related quality of life (EQ-5D), and whether the individual has private health insurance (yes, no). Obesity status was determined using Body Mass Index (BMI) data collected during the same year as the index hospitalization [21]. Health-related quality of life was measured using the EQ-5D index, where a score in the bottom 25% of all respondents from the 2019 Korean Medical Panel was classified as “poor,” and above this threshold as “good” [22]. Specifically, an EQ-5D index score of 0.9338 or higher was classified as “good,” while scores between −0.5944 and 0.9338 were classified as “poor” [23]. The selection of these specific demographic, socioeconomic, and clinical covariates was informed by established evidence identifying them as significant predictors of healthcare utilization trajectories and financial burden in South Korea [19,20,24].

#### 2.4. Statistical analysis.

This study examined the healthcare utilization patterns of musculoskeletal surgery patients after discharge and analyzed the factors affecting healthcare expenditures in the first and second years post-discharge. First, trends in healthcare utilization (total healthcare expenditures, inpatient care, outpatient care in WM, and outpatient care in TKM at 6-month, 12-month, 18-month p.12, and 24-month intervals were observed and visualized. Multiple linear regression analysis was conducted to identify the factors influencing healthcare expenditures and high-expenditure in the second year post-discharge were identified. Information on the healthcare utilization information (inpatient care, outpatient care in western medicine, and outpatient care in TKM) in the first year after discharge was included to predict the 2-year post-discharge utilization. The regression analysis was stratified into two models to examine TKM utilization at different levels of granularity. Model 1 utilized the total number of TKM outpatient visits to assess general utilization impact, while Model 2 replaced this with specific counts for each TKM modality—acupuncture, herbal medicine, and chuna/manual therapy—to isolate the effects of different therapeutic goals on second-year costs. The goodness-of-fit for the regression models was evaluated using the coefficient of determination (R-square), representing the explained variance [25], and the Akaike Information Criterion (AIC) for model comparison [26]. Model 1 yielded an R-square of 0.244 and an AIC of 200.428. Model 2, which disaggregated Traditional Korean Medicine utilization, demonstrated a slightly superior fit for the data with an R-square of 0.245 and a lower AIC of 199.152.

The covariates, such as demographic, socioeconomic, behavioral and clinical conditions, were adjusted to minimize potential confounding in the regression models. We utilized multivariable regression models that adjusted for a broad range of potential confounders, including age, residential area, income level, and multimorbidity, which are known to influence both healthcare utilization and expenditure patterns in the South Korean context [18,19]. All analyses were performed using SAS Software 9.4.

#### 2.5. Ethics statement.

The original data are publicly available free of charge from the KHP website (https://www.khp.re.kr) for the purposes of academic research. Due to the retrospective nature of this study, which utilized data with encrypted personal information, it was exempted from ethical approval by the Public Institutional Review Board (IRB) Designated by Ministry of Health and Welfare (No. P01-202309-01-034). All authors read and followed the tenets of the Declaration of Helsinki in preparing this study.

## 3. Results

### General characteristics of the study participants

Among 182 patients (65.4% women, mean age 64 years old), 21.4% lived in the capital area. The study population consisted primarily of National Health Insurance beneficiaries (94.5%), with Medical Aid recipients making up the remaining 5.5%. In terms of employment, 25.8% of participants were wage workers, while 28.0% were self-employed. During the previous year, 48.9% exercised regularly, 9.3% smoked, 17.6% binge-drank, and 42.3% were obese (BMI ≥ 25 kg/m²). Patients averaged 2.4 chronic diseases; 45.1% held indemnity insurance and 55.5% reported poor EQ-5D quality of life.

Index admissions for musculoskeletal surgery involved spinal disorders at 42.3%, knee osteoarthritis 30.2%, shoulder conditions 14.3%, and other joint or fracture-related problems 13.2%. Operations took place in metropolitan facilities for 33.5% of cases, exceeding the residential proportion. Most surgeries were performed in hospitals (53.9%), followed by general or tertiary hospitals (36.8%) and clinics (9.3%). Mean length of stay was 14.1 days and the mean admission charge was about KRW 3.11 million (≈ US $2 630, 2020 rate). Post-discharge, patients initially sought care at hospitals (36.8%), general or tertiary hospitals (29.7%), or clinic-level facilities (29.1%) or not at all (4.4%). Clinic visits and non-utilization were combined as “community-based follow-up” for regression analyses (Table 1).

**Table 1:** General characteristics of the study participants (N = 182).

Variable	Category	n	%
Sex	Male	63	34.6
	Female	119	65.4
Age, years	Mean ± SD (range)	64 ± 12.4 (19–85)	
Residential area	Metropolitan area	39	21.4
	Non‑metropolitan area	143	78.6
Household‑income quartile	Q1 (lowest)	45	24.7
	Q2	46	25.3
	Q3	45	24.7
	Q4 (highest)	46	25.3
Marital status	Unmarried	53	29.1
	Married	129	70.9
Type of health insurance	National Health Insurance	172	94.5
	Medical aid	10	5.5
Job status	Economically inactive	84	46.2
	Wage worker	47	25.8
	Employer / self‑employed	51	28.0
Regular exercise (past 12 month)	No	93	51.1
	Yes	89	48.9
Current smoking	No	165	90.7
	Yes	17	9.3
Binge drinking (past 12 month)	No	150	82.4
	Yes	32	17.6
Obesity	BMI < 25 kg m²	105	57.7
	BMI ≥ 25 kg m²	77	42.3
Number of chronic conditions	Mean ± SD (range)	2.4 ± 1.5 (0–7)	
Private indemnity insurance	No	100	55.0
	Yes	82	45.1
Health‑related QoL (EQ‑5D)	Good	81	44.5
	Poor	101	55.5
Characteristics of index hospitalization			
Principal diagnosis	Spinal disorders	77	42.3
	Knee osteoarthritis	55	30.2
	Shoulder disorders	26	14.3
	Other joint / muscle pain, fractures, gout	24	13.2
Hospital location	Metropolitan area	61	33.5
	Non‑metropolitan area	121	66.5
Facility type	Clinic	17	9.3
	Hospital	98	53.9
	General / tertiary hospital	67	36.8
Length of stay, days	Mean ± SD	14.1 ± 11.9	
Total admission charge (KRW, USD)	Mean ± SD	3,116,427 ± 2,581,362 (USD: 2,630 ± 2,180)	
First post‑discharge pathway	No	8	4.4
	Clinic‑level	53	29.1
	Hospital	67	36.8
	General / tertiary hospital	54	29.7
Total		182	100.0

Note. Amounts in parentheses were converted to 2020 US dollars using the 2020 annual average exchange rate (1 USD = 1 184.1 KRW; Bank of Korea/X‑Rates, 2020).

### Two-year post-discharge healthcare utilization among patients with musculoskeletal disorders

The musculoskeletal readmission rate decreased across four consecutive 6-month intervals: from 13.2% (0–6 months) to 7.7% (6–12 months), 4.9% (12–18 months), and 2.7% (18–24 months). Inpatient care costs were less consistent, averaging USD 923.4 in the first half-year, USD 2454.7 at 6–12 months, USD 1270.5 at 12–18 months, and USD 2183.7 at 18–24 months. Outpatient utilization similarly declined over these periods, falling from 80.2% in the first six months to 78.0%, 69.2%, and 59.9% in the subsequent intervals, with corresponding mean charges of USD 360.2, 224.6, 263.4, and 227.7. Overall service use (inpatient + outpatient) fell from 82.4% in months 0–6 to 81.9%, 74.2%, and 63.7% in successive half-year periods; mean combined charges decreased from USD 557, 475, 367, 337.

**Fig 2:**
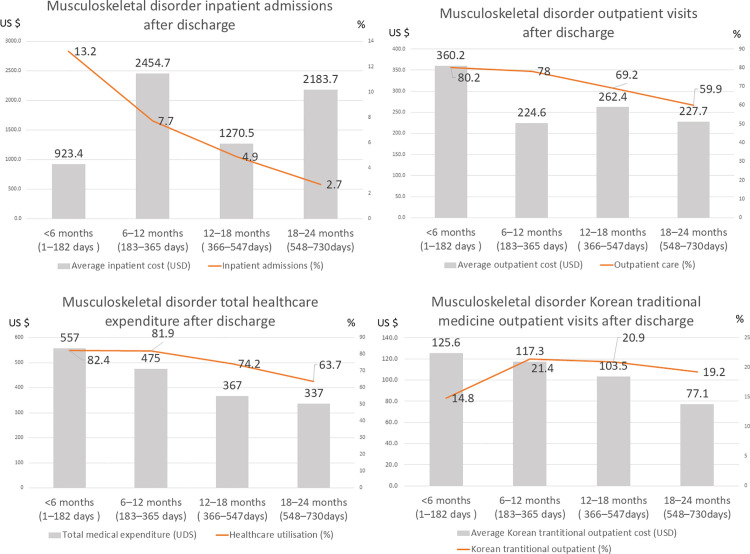
Post-discharge patterns of healthcare for musculoskeletal disorder.

In contrast, TKM outpatient visits increased after the initial recovery period: utilization rose from 14.8% (0–6 months) to 21.4% at 6–12 months, 20.9% at 12–18 months, and 19.2% at 18–24 months. Despite the higher visit rate, mean TKM outpatient charges declined steadily to USD 125.6, 117.3, 103.4, and 77.1 across the four intervals. Collectively, these data show that while TKM utilization and costs diminish over two years, roughly one-fifth of patients continue to seek TKM consultations during longer-term recovery (Fig 2).

**Fig 3:**
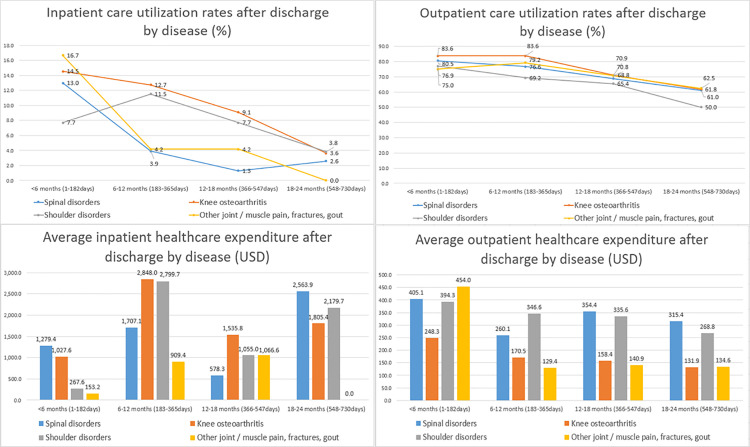
Comparative longitudinal trajectories of post-discharge healthcare utilization and expenditures across musculoskeletal diagnostic groups over 24 months.

Diagnosis-specific analysis revealed distinct trajectories in healthcare utilization and spending across the four groups. Inpatient care utilization rates generally declined over 24 months, though knee osteoarthritis patients maintained the highest rates of readmission during the 6–12 month (12.7%) and 12–18 month (9.1%) intervals compared to other conditions. Despite declining utilization rates, average inpatient healthcare expenditures showed significant late-stage spikes; notably, spinal disorder expenditures reached USD 2,563.9 in the 18–24 month period, while shoulder disorders peaked at USD 2,799.7 between 6 and 12 months post-discharge. Outpatient care utilization rates remained high for all groups initially, but knee osteoarthritis patients exhibited the most persistent engagement, with 83.6% still utilizing outpatient services one year after discharge. Finally, average outpatient healthcare expenditures remained consistently elevated for spinal and shoulder disorders throughout the two-year period, whereas expenditures for the other joint/muscle pain and fractures group dropped sharply from USD 454.0 in the first six months to USD 134.6 by the end of the follow-up (Fig 3).

### Post-discharge healthcare utilization during the first and second years following the index hospitalization among patients with musculoskeletal disorders

During the first post-discharge year (days 1–365) mean total expenditure was KRW 1 004 255 (≈ US $848), falling to KRW 576 764 (≈ US $487) in days 366–730. The high-cost threshold (top 25%) likewise dropped from KRW 1 017 360 (≈ US $859) to KRW 551 700 (≈ US $466). The first year readmission reached 19.2%, with averages of 18.3 WM outpatient visits and 2.3 TKM visits. TKM use was led by acupuncture/moxibustion/cupping/pharmacopuncture (3.5 sessions), followed by chuna/manual/physiotherapy (2.3) and herbal formulations (0.4).

In the second year, while the readmission rate dropped to 7.1%, the mean frequency of Western Medicine (WM) outpatient visits was 13.6 ± 22.9, and the mean frequency of Traditional Korean Medicine (TKM) visits rose to 3.3 ± 10.0 per patient. Session frequencies also shifted: acupuncture-type treatments rose to 4.6 and chuna/manual/physiotherapy to 3.3, while herbal therapy remained low at 0.3. These trends indicate a marked reduction in overall utilization and costs after the first year, accompanied by a modest but sustained rise in TKM engagement, with TKM visits increasing from 2.3 in the first year to 3.3 in the second year (Table 2). Amounts in parentheses are 2020-USD equivalents (1 USD = 1 184.1 KRW).

**Table 2:** Post-discharge healthcare utilization during the first and second years following the index hospitalization among patients with musculoskeletal disorders.

Outcome	The first year after discharge (1–365 days)	The second year after discharge (366–730 days)
Total medical expenditure (KRW) (USD)	1,004,255 (USD: 848)	576,764 (USD: 487)
High-expenditure (top 25%)	46 (25.3%)	46 (25.3%)
Threshold amount (KRW, USD)	1,017,360 (USD: 859)	551,700 (USD: 466)
Hospital admissions – patients admitted, n (%)	35 (19.2%)	13 (7.1%)
Admissions per patient, mean ± SD	0.26 ± 0.72	0.08 ± 0.31
Out‑patient visits – WM, mean ± SD	18.3 ± 25.9	13.6 ± 22.9
Out‑patient visits – TKM total, mean ± SD	2.3 ± 6.5	3.3 ± 10.0
Acupuncture/moxibustion/cupping/pharmacopuncture	3.5 ± 10.9	4.6 ± 14.8
Decoction/expensive formulas/herbal medicine	0.4 ± 2.5	0.3 ± 1.4
Chuna/manual therapy/physiotherapy	2.3 ± 6.4	3.3 ± 10.1
Total	182	(100.0%)

Note. Amounts in parentheses were converted to 2020 US dollars using the 2020 annual average exchange rate (1 USD = 1 184.1 KRW; Bank of Korea/X‑Rates, 2020).

TKM, Traditional Korean Medicine; WM, Western Medicine.

### Factors associated with the second-year expenditure and high-expenditure after index hospitalization

Determinants of second-year expenditure were examined in two models. Model 1 used the total number of TKM outpatient visits during year 1, Model 2 replaced this with counts for each TKM modality. In the Model 1, metropolitan residence, obesity, and more chronic conditions all increased the likelihood of high-expenditure in the second-year after index hospitalization. Conversely, lower income (Q1–Q3 vs Q4) predicted lower costs. Index admission also mattered: spinal or shoulder diagnoses, heavier inpatient use during the first year after index hospitalization, increased later expenditure in the second year. In the Model 2, when TKM care was disaggregated, only the frequency of chuna/manual therapy/physiotherapy marginally increased the odds of becoming a high-cost patient in year 2 (OR = 1.31, 95% CI 1.00–1.71), while sociodemographic factors, index diagnoses, and prior service use mirrored those in Model 1 (Table 3).

**Table 3:** Factors associated with the second-year expenditure and high-expenditure after index hospitalization.

		Model 1		Model 2	
		log(Healthcare expenditure)	High-expenditure	log(Healthcare expenditure)	High-expenditure
		β (SE)	OR (95% CI)	β (SE)	OR (95% CI)
Sex (ref. = Female)	Male	–0.636 (0.961)	1.07 (0.36–3.21)	–0.618 (0.968)	1.04 (0.34–3.21)
Age (yrs.)	(Continuous)	0.067 (0.047)	1.01 (0.95–1.06)	0.069 (0.047)	1.01 (0.95–1.07)
Residential area (ref. = Non‑metropolitan)	Metropolitan	1.504 (1.444)	6.60* (1.24–35.21)	1.497 (1.455)	6.82* (1.21–38.58)
Household‑income quartile (ref. = Q4)	Q1	–1.997 (1.369)	0.14* (0.03–0.69)	–2.117 (1.410)	0.11* (0.02–0.61)
	Q2	–2.451* (1.237)	0.19* (0.05–0.75)	–2.573* (1.287)	0.16* (0.04–0.69)
	Q3	–1.870 (1.152)	0.25* (0.07–0.95)	–1.994 (1.183)	0.20* (0.05–0.83)
Marital status (ref. = Unmarried)	Married	−0.417 (0.955)	1.48 (0.48-4.54)	−0.428 (0.966)	1.70 (0.52-5.55)
Type of health insurance (ref. = Medical aid)	National Health Insurance	2.651 (1.818)	0.35 (0.03-3.78)	2.731 (1.842)	0.46 (0.04-4.83)
Job status (ref. = Employer/ self‑employed)	Economically inactive	0.962 (0.984)	0.58 (0.20-1.73)	1.018 (1.002)	0.60 (0.19-1.89)
	Wage worker	1.427 (1.127)	0.46 (0.12-1.73)	1.449 (1.134)	0.41 (0.10-1.67)
Regular exercise (ref. = No)	Yes	0.395 (0.855)	0.42 (0.16–1.12)	0.340 (0.867)	0.42 (0.15–1.17)
Current smoking (ref. = No)	Yes	–2.838 (1.629)	0.17 (0.02–1.66)	–2.803 (1.640)	0.20 (0.02–2.02)
Binge drinking (ref. = No)	Yes	–1.561 (1.177)	0.20 (0.04–1.05)	–1.564 (1.185)	0.21 (0.04–1.22)
Obesity (ref. = BMI < 25 kg m²)	BMI ≥ 25 kg m²	–0.356 (0.823)	3.52* (1.33–9.34)	–0.380 (0.829)	3.22* (1.20–8.66)
Private indemnity insurance (ref. = No)	Yes	0.332 (0.980)	1.21 (0.39-3.76)	0.317 (0.996)	0.99 (0.30-3.23)
Number of chronic conditions	(Continuous)	0.400 (0.321)	1.07 (0.76-1.52)	0.395 (0.323)	1.08 (0.76-1.55)
Health‑related quality of life (EQ‑5D) (ref. = Good)	Poor	0.326 (0.917)	0.97 (0.35-2.69)	0.331 (0.924)	1.11 (0.39-3.12)
[Characteristics of index hospitalization]					
Principal diagnosis (ref. = Other joint / muscle pain, fractures, gout)	Spinal disorders	0.842 (1.234)	7.55* (1.30-43.99)	0.819 (1.249)	6.78* (1.13–40.76)
	Knee osteoarthritis	0.156 (1.343)	2.54 (0.39-16.38)	0.211 (1.355)	2.55 (0.39-16.90)
	Shoulder disorders	0.920 (1.525)	9.23* (1.23-69.45)	0.892 (1.548)	7.14 (0.90-56.65)
Hospital location (ref. = Non‑metropolitan area)	Metropolitan area	−0.084 (1.254)	0.73 (0.17-3.14)	−0.154 (1.268)	0.58 (0.13-2.62)
Facility type (ref. = General / tertiary hospital)	Clinic	1.270 (1.454)	2.80 (0.60-12.98)	1.217 (1.474)	2.72 (0.56-13.10)
	Hospital	−0.196 (0.863)	1.71 (0.61-4.80)	−0.256 (0.872)	1.42 (0.49-4.13)
[Post-discharge healthcare utilization during the first year after index hospitalization]					
Number of admissions		0.028 (0.015)	1.02* (1.00–1.04)	0.029 (0.016)	1.02* (1.00–1.05)
Number of outpatient visits		1.254* (0.558)	2.00 (0.91–4.36)	1.253* (0.562)	2.17 (0.92–5.13)
Number of total TKM outpatient visits		0.095 (0.062)	1.03 (0.96-1.10)		
Number of Acupuncture, moxibustion, cupping, pharmacopuncture				−0.003 (0.091)	0.87 (0.73-1.03)
Number of Decoction, high-cost herbal formulas, general herbal medicine				−0.099 (0.182)	0.80 (0.54-1.17)
Number of Chuna therapy, manual therapy, physiotherapy				0.118 (0.166)	1.31 (1.00-1.71)
Model fit		R-Square: 0.244	AIC: 200.428	R-Square: 0.245	AIC: 199.152

SE, standard error; OR, odds ratio; CI, confidence interval. *p < 0.05.

TKM, Traditional Korean Medicine; WM, Western Medicine.

## 4. Discussion

This study utilized data from the KHP (2019–2021) to examine changes in healthcare utilization over two years among patients who were hospitalized for musculoskeletal disease-related surgeries. Additionally, the study examined factors associated with healthcare expenditure and high-cost cases (top 25%) in second year after discharge among patients hospitalized for musculoskeletal surgery between July 2018 and June 2020.

The results of tracking healthcare utilization for two years in six-month intervals after discharge show a trend of decreasing overall healthcare utilization over time. This suggests that, while patients initially rely heavily on medical services following surgery, the demand for such services gradually declines as recovery progresses [27]. Both inpatient admissions and outpatient visits decreased over time. However, in contrast to this general trend, the rate of TKM outpatient visits increased after the first six months post-surgery. This suggests that patients, particularly during the stabilization and rehabilitation phases, may increasingly incorporate diverse treatment options, including TKM, for pain management and functional recovery [28,29].

In the second year after discharge, both the primary diagnosis at index hospitalization and the healthcare utilization patterns during the first post-discharge year significantly influenced the likelihood of becoming a high-cost patient. Specifically, patients whose index diagnoses involved spinal or shoulder disorders were more likely to fall into the top 25% of medical spenders in the second year, consistent with prior research showing that such conditions often require prolonged rehabilitation and lead to higher long-term expenditures [30]. While high inpatient or outpatient utilization in the first year post-discharge was associated with continued high medical costs in the second year, this finding raises questions about whether early intensive intervention reduces or inadvertently sustains long-term expenditures—a pattern observed in hypertension trajectory studies [11], musculoskeletal pain research [31], high-need high-cost patient analyses [32], and Korean elderly healthcare spending trajectories [33] where initial high utilization predicted persistent high-cost groups.

The observed increase in TKM utilization during the second year post-discharge reflects a shift in patient needs from acute surgical management to long-term functional rehabilitation and pain control [12]. TKM complements Western Medicine by providing non-invasive therapeutic modalities, such as acupuncture for pain relief and chuna therapy for musculoskeletal correction, which address lingering symptoms during the stabilization phase [34,35]. This transition suggests that patients increasingly view TKM as a primary pathway for holistic recovery when the focus shifts from structural repair to functional restoration in the community [10]. Integrating these complementary approaches aligns with South Korea's “Integrated Community Care” initiatives, which emphasize seamless continuity of care between hospital-based acute treatment and community-based rehabilitation [36].

In addition, a disaggregated analysis of TKM modalities showed that frequent use of chuna therapy, manual therapy, and physical therapy during the first year was marginally associated with an increased likelihood of becoming a high-cost patient in the second year. This finding contrasts with previous studies suggesting that such TKM reduce long-term healthcare costs by promoting recovery [37–39]. The TKM treatments were suggested to be cost-effective for certain disorders [18,34]. The observed associations may be influenced by higher overall healthcare utilization, rather than reflecting the inherent cost-effectiveness of the therapies themselves. One possible explanation is that these modalities may be more commonly used by patients with persistent or severe postoperative symptoms, thus serving as a proxy for underlying health complexity rather than inefficiency [35]. For example, Chuna therapy is significantly more effective than usual care in reducing pain and functional disability [40]. Furthermore, electroacupuncture has proven to be highly cost-effective for post-surgical pain, with an incremental cost-effectiveness ratio (ICER) of KRW 7.05 million per QALY, well below the national threshold of KRW 30 million [41]. These findings suggest that higher short-term TKM expenditures reflect value-driven care that enhances long-term quality of life. Therefore, policy-makers need to prioritize establishing standardized protocols to optimize TKM utilization within integrated care pathways rather than simply restricting access to control costs.

Beyond clinical factors, individual sociodemographic and behavioral characteristics significantly shaped second-year expenditures. The inclusion of socioeconomic factors highlights that higher income levels may facilitate greater access to elective or supportive services, whereas lower-income groups may face barriers to comprehensive rehabilitation, thus influencing the total expenditure observed [18-20]. These findings align with prior studies demonstrating that poor health status and higher body mass index are positively correlated with increased healthcare expenditures [20,24]. Patients residing in metropolitan areas, along with those who are obese or have multiple chronic conditions, were found to consistently incur higher medical costs [19]. This significant association between metropolitan residence and higher expenditure is largely driven by the disproportionate concentration of tertiary general hospitals and specialized medical centers in these regions, which typically charge higher treatment fees and co-payments compared to facilities in non-metropolitan areas. Furthermore, the superior physical accessibility to high-cost medical infrastructure in metropolitan cities often encourages more frequent and intensive healthcare utilization, including medical migration from rural areas, thereby exacerbating the overall financial burden on patients [19]. In a broader context, these geographic and metabolic drivers of expenditure mirror global trends where obesity and urban health infrastructure disproportionately influence medical spending [42,43]. This study provides the necessity of evidence-based protocols that define the timing and frequency of TKM interventions. Establishing standardized care pathways is essential for ensuring clinical continuity and economic efficiency, particularly in mitigating the healthcare disparities observed across different residential and socioeconomic groups.

This study has several limitations. First, the sample of patients hospitalized for musculoskeletal diseases in the Korean Health Panel was small, which may limit the representativeness and generalizability of the findings. Second, key factors such as surgical complexity, complications, and caregiving or nursing support, which may influence post-discharge medical use, were not included. Third, the study period (2019–2021) coincided with the COVID-19 pandemic in South Korea, during which healthcare utilization patterns were substantially disrupted, however, the impact of COVID-19 could not be precisely assessed. Finally, using the number of inpatient and outpatient visits as proxies for the continuity of post-discharge care may not fully capture ongoing management. Incorporating variables such as the use of community-based services (e.g., commercial rehabilitation centers) would yield a more comprehensive assessment. Future research should incorporate more detailed clinical and contextual information, including surgical complexity, postoperative complications, and the availability of caregiving or nursing support, to better capture factors influencing post-discharge healthcare utilization [44,45]. Integrating these variables would allow for a more comprehensive understanding of recovery trajectories and continuity of care following hospitalization for musculoskeletal conditions.

Despite these limitations, the policy implications of this study are as follows. Given that healthcare expenditures are concentrated within the first 6 to 12 months after musculoskeletal surgery, with increased TKM utilization showing cost-neutral effects through reduced rehospitalization, while chuna/manual/physiotherapy exhibits marginal cost increases policies must target value-based integration rather than unrestricted expansion of these modalities. For instance, expanding support for community-based rehabilitation and outpatient TKM services, alongside financial protection mechanisms like bundled payments for high-risk patients could alleviate the economic burden on patients and prevent health deterioration, contributing to long-term cost savings.

From a health-security perspective, buffering early post-discharge cost surges addresses not only individual affordability but also national capacity by curbing avoidable readmissions via coordinated Western-TKM pathways with shared protocols and risk stratification [9,46]. Furthermore, since health behaviors like obesity and chronic conditions affect high-expenditures, community lifestyle programs integrated with tailored post-discharge care—emphasizing early TKM for pain management and physiotherapy optimization—can reduce utilization while enhancing functional recovery [44,47,48].

This study clarifies post-discharge healthcare trajectories for musculoskeletal disorders, underscoring that simply increasing usage is insufficient. Instead, stratified and coordinated Western-TKM care pathways or integrating early risk stratification are essential to inform financial protection strategies for high-risk patients, mitigating catastrophic expenditures while optimizing long-term outcomes.

## 5. Conclusions

Patients with musculoskeletal disorders showed a decreasing trend in healthcare utilization and expenditures over time. In the second year post-discharge, spinal and shoulder disorders, as well as the number of admissions and outpatient visits during the first year after discharge, were associated with higher healthcare expenditures. These findings underscore the importance of early risk stratification and tailored, coordinated care pathways to support effective post-discharge management, rather than relying solely on increasing the volume of services. For healthcare providers and policymakers, proactive and integrated care strategies linking inpatient treatment with timely follow-up and supportive services may help improve continuity of care. Future studies using larger-scale or multi-center data are warranted to enhance generalizability and to further examine post-discharge care patterns across diverse healthcare settings.
